# Potentials of *Bacillus subtilis-*derived surfactin to improve performance, intestinal health and welfare of broilers under necrotic enteritis challenge

**DOI:** 10.1016/j.psj.2025.106091

**Published:** 2025-11-10

**Authors:** Alip Kumar, Kosar Gharib-Naseri, Most Khairunnesa, Sosthene Musigwa, Reza Barekatain, Li Li, Peng Chen, Shu-Biao Wu

**Affiliations:** a School of Environmental and Rural Science, University of New England, Armidale, NSW 2351, Australia; b SARDI Roseworthy campus, University of Adelaide, SA-5371, Australia; c Beijing Enhalor International Tech Co. Ltd., Beijing, China

**Keywords:** Surfactin, Performance, Intestinal health, Necrotic Enteritis, Broiler chicken

## Abstract

This study evaluated the potential of *Bacillus subtilis*-derived surfactin (**SRF**) on broiler performance, intestinal health and welfare parameters under necrotic enteritis (**NE**) challenge. A total of 512 d-old Cobb 500 broiler chicks were allocated into four treatment groups, with eight replicates each. The treatments were: **NC**, non-challenged control; **CC**, NE challenged control; SRF, CC + surfactin at 0.01 %; and **ANT**, CC + antibiotics: zinc bacitracin and salinomycin across all feeding phases. Birds were fed a wheat-soybean meal-sorghum-based diet supplemented with xylanase and phytase. The NE challenge was induced by oral gavage of *Eimeria* spp. on d 9 and NetB-producing *Clostridium perfringens* on d 14 and 15. The results showed that before the challenge, average weight gain (**AWG**), average feed intake (**AFI**) and feed conversion ratio (**FCR**) were not different among the treatments (*P* > 0.05). During post-NE challenge (d 8-19) and overall period (d 0-35), AWG and FCR were compromised by NE, with increased intestinal lesions and serum fluorescence isothiocyanate dextran (**FITC-d**) concentrations on d 16 in the CC group than NC group (*P* < 0.05), indicating a successful sub-clinical NE challenge. Birds fed ANT had higher AWG and lower FCR, intestinal lesions and serum FITC-d concentrations than the CC group (*P* < 0.05). Supplementation of SRF significantly improved FCR (*P* < 0.05) during d 28-35, while during the overall period (d 0-35), SRF-fed birds showed AWG and production efficiency factor (**PEF**) that shifted towards those of the NC, being not significantly different (*P* > 0.05). Birds fed SRF had increased jejunal villus height and crypt depth ratio (**VH:CD**), reduced duodenal lesion and footpad dermatitis scores than CC group (*P* < 0.05). Additionally, SRF supplementation shifted serum FITC-d, litter moisture, litter, hock burn and jejunal lesion scores towards the levels comparable to NC and ANT groups (*P* > 0.05). These findings suggest that surfactin may have the potential to support recovery from the NE, enhancing performance, intestinal health, and overall welfare in broilers.

## Introduction

Necrotic enteritis (**NE**) is an enteric bacterial disease of poultry mainly caused by NetB-producing Clostridium perfringens, a spore-forming, Gram-positive, anaerobic, universal bacterium, along with predisposing factors such as dietary ingredients (e.g., fish meal) and pathogens (e.g. *Eimeria* spp.) (Keyburn et al., 2008; Moore, 2016). The sub-clinical form of NE can lead to impaired performance, increased intestinal lesions, diarrhoea and wet litter, while the clinical form of NE is marked by sudden deaths and significantly high flock mortality (Kaldhusdal, et al., 2001; Immerseel, et al., 2004). The global poultry industry incurs an estimated annual cost exceeding US$6 billion due to NE, attributed to impaired performance, high mortality and costs related to disease control and management (Wade and Keyburn, 2015). The disease can impair the intestinal villi development, mucosa and epithelial cells (Immerseel, et al., 2004; Palliyeguru and Rose, 2014), resulting in intestinal lesions, inflammation and alteration of the intestinal microbial population (Gharib-Naseri, et al., 2019). It also disrupts tight junction proteins, impairing gut barrier function (Latorre, et al., 2018). As a result, birds infected with NE exhibit increased intestinal permeability and reduced growth performance (Awad, et al., 2017). In-feed antibiotics had been commonly used to control NE in poultry. However, their use has been banned or restricted globally due to the rise of antibiotic-resistant bacteria, which pose significant risks to animal and human health, leading to a higher incidence of NE (Kocher and Choct, 2008; Kaldhusdal, et al., 2016). This shift has driven the search for alternative solutions, leading to the introduction of various additives in the poultry industry, including probiotics, prebiotics, phytogenics and organic acids.

The utilisation of probiotics has emerged as a prominent method in recent years (Shini and Bryden, 2021), with increasing data indicating their substantial efficacy in preventing NE (Khalique, et al., 2020; Wang, et al., 2021). Bacillus spp. strains are the most promising probiotic feed supplements for poultry, as their spores demonstrate health-promoting benefits and the ability to endure extreme environmental conditions, including high temperatures during pelleting and low pH in the gastrointestinal tract (Shivaramaiah, et al., 2011). Bacillus spores enhance gut health by competitive exclusion, synthesis of antimicrobial peptides and beneficial metabolites, and activation of the intestinal immune system (Hayashi, et al., 2018). *Bacillus subtilis* exhibits extensive efficacy against Clostridium spp. and enhances overall performance in broilers (Jayaraman, et al., 2013). Antimicrobial peptides generated from bacteria, specifically extracted from *B. subtilis*, exhibit a wide range of antimicrobial efficacy against pathogenic microorganisms (Sumi, et al., 2015). Surfactin (**SRF**), a cyclic lipopeptide from the non-ribosomal peptide family, is a significant biosurfactant known for its outstanding emulsifying abilities and its antibacterial, antiviral, anticancer, and anti-inflammatory (Vollenbroich, et al., 1997; Heerklotz and Seelig, 2001; Chen, et al., 2008), wound healing and scar inhibitory properties (Yan, et al., 2020). A recent study showed the positive effects of SRF on weight gain, tight junction proteins and microbiota composition under a cyclophosphamide-induced gut dysbiosis model in mice (Jia, et al., 2024). Another study reported that B. subtilis fermented product enriched with SRF significantly improved performance, histomorphology and bone health (Lee, et al., 2024). However, the effect and underlying mechanisms of SRF in mitigating the adverse impact of NE on performance and intestinal health, and improving the bird welfare, are yet to be explored.

It was hypothesised that dietary SRF supplementation mitigates the negative effects associated with NE on intestinal health and improves the performance and welfare of broilers. Therefore, this study was designed to evaluate the effect of SRF on performance, intestinal health and bird welfare under NE challenge.

## Materials and methods

### Animal ethics statement

The experimental procedures applied in the present study were reviewed and approved by the Animal Ethics Committee of the University of New England, Armidale, NSW 2351, Australia (AEC 23-003).

### Birds and housing management

A total of 512 d-old Cobb 500 mixed-sex chicks were obtained from Baiada hatchery in Tamworth, NSW, Australia. Upon arrival, birds were weighed and randomly assigned to four treatments with approximately equal total weight in 32-floor pens measuring 120 × 77 cm, using a completely randomised design. Each of the four treatments comprised eight replicate pens with 16 birds per pen at the start. Birds were reared in a climate-controlled environment with softwood shavings as bedding material. Each pen had three nipple drinkers and a feeder, and feed and water were supplied *ad libitum*. Lighting, relative humidity, and temperature were maintained in accordance with Cobb 500 standard guidelines (Cobb500, 2022b).

### Experimental design and dietary treatments

The current study comprised four treatment groups in a completely randomised design, one non-challenged group as the control and three groups subjected to NE challenge. The aim was to evaluate the efficacy of SRF, SURFA TID® provided by Beijing Enhalor International Tech Co. Ltd., China, a fermented feed additive comprising *B. subtilis* spores and the cyclic lipopeptide produced from the bacterium, with SRF as a primary active ingredient, in broilers under a NE challenge**.** The treatments were: **NC**, non-challenged control, without additives or antibiotics; **CC**, challenged control, without additives or antibiotics; **SRF**, challenged group supplemented with surfactin at 0.01 %; **ANT**, challenged group with antibiotics: zinc bacitracin and salinomycin at 0.027 % and 0.05 % in starter, grower and finisher phases (Table 1). Experimental diets were formulated with wheat, soybean meal and sorghum as major ingredients, where matrix values of phytase (1000 FTU/kg) were considered in the diet formulation as shown in Table 2. Prior to feed formulation, near-infrared spectroscopy (AminoNIR®, Evonik Operations GmbH, Essen, Germany) was used to determine nutrient contents of feed ingredients and the NIR values are shown in Table 3. Diets were cold pelleted and provided *ad libitum* in three phases: starter phase (d 0-8; crumbled), grower phase (d 8-19), and finisher phase (d 19-35) following Cobb 500 nutrient specifications and feeding standards for broilers in each phase (Cobb500, 2022a).

**Table 1:** Treatment groups with additives applied in this study.

Treatments^1^	Additives	Inclusion level, %; all phases	Necrotic enteritis challenge^2^
NC	-	-	Non-challenged
CC	-	-	Challenged
SRF	Surfactin	0.01	Challenged
ANT	Antibiotics	Zn bacitracin: 0.027 + Salinomycin: 0.050	Challenged

Zn = Zinc.

1 NC, Non-challenged control; CC, Challenged control; SRF, Surfactin; ANT, Antibiotics (Zn bacitracin and Salinomycin).

2 Challenged birds were orally gavaged with *Eimeria* spp. on d 9 and *Clostridium perfringens* on d 14 and 15.

**Table 2:** Experimental diet and calculated nutrient composition (as-fed basis, % unless declared otherwise).

Ingredients, %	Starter phase (d 0-8)	Grower phase (d 8-19)	Finisher phase (d 19-35)
Wheat	46.5	52.2	57.9
Soybean meal	31.5	26.3	20.2
Sorghum	14.7	13.8	15.0
Canola oil	3.25	3.40	3.00
Limestone	1.07	1.13	1.09
Dicalcium phosphate	0.973	0.460	0.323
L-methionine	0.353	0.366	0.350
L-lysine HCl	0.347	0.380	0.455
Salt	0.230	0.240	0.220
L-threonine	0.180	0.180	0.155
L-Arginine HCl	0.145	0.195	0.340
L-Valine	0.114	0.145	0.172
Vitamin premix^1^	0.080	0.080	0.080
Mineral premix^2^	0.080	0.080	0.080
Choline chloride 60 %	0.062	0.082	0.102
Sodium bicarbonate	0.054	0.051	0.082
L-Isoleucine	0.010	0.064	0.108
Xylanase^3^	0.016	0.016	0.016
Phytase^4^	0.020	0.020	0.020
Titanium dioxide	0.000	0.500	0.000
Sand^5^	0.293	0.407	0.281
**Calculated nutrient composition^6^**			
Dry Matter	89.1	89.0	88.7
AMEn, kcal/kg	3,005	3,050	3,100
Crude protein	22.2	20.4	18.6
Crude fat	4.83	4.98	4.63
Crude fiber	2.87	2.77	2.69
Digestible Arg	1.38	1.28	1.24
Digestible Lys	1.26	1.16	1.08
Digestible Met	0.628	0.616	0.574
Digestible Met + Cys	0.949	0.917	0.853
Digestible Trp	0.286	0.260	0.232
Digestible Ile	0.850	0.812	0.756
Digestible Thr	0.903	0.827	0.719
Digestible Val	0.985	0.930	0.865
Calcium	0.900	0.800	0.740
Phosphorus available	0.500	0.400	0.370
Sodium	0.180	0.180	0.180
Potassium	0.967	0.872	0.770
Chloride	0.255	0.272	0.280
Choline, mg/kg	1,750	1,750	1,744
Linoleic 18:2	1.45	1.50	1.41

AMEn = apparent metabolisable energy, nitrogen-corrected.

1 Vitamin premix provided the following per kilogram diet: vitamin A, 12 MIU; vitamin D, 5 MIU; vitamin E, 75 mg; vitamin K, 3 mg; cyanocobalamin, 0.016 mg; folic acid, 2 mg; riboflavin, 8 mg; pyridoxine, 5 mg; biotin, 0.25 mg; thiamine, 3 mg; nicotinic acid, 55 mg; pantothenic acid, 13 mg and antioxidant ethoxyquin, 50 mg.

2 Mineral premix provided the following per kilogram diet: Cu sulfate, 16 mg; Mn sulfate, 60 mg; Mn oxide, 60 mg; I (iodide), 0.125 mg; Se (selenite), 0.3 mg; Fe sulfate, 40 mg; Zn oxide and sulfate, 100 mg.

3 Xylanase: Rovabio® at 4000 VU/kg feed (160 g/t). ^4^Phytase: Quantum Blue 5 G at 1000 FTU/kg (200 g/t).

5 Sand was replaced with the required amount of additives. ^6^Ingredients were analysed using near-infrared spectroscopy.

**Table 3:** Analysed nutrient composition of ingredients (%, unless declared otherwise).

NIR analysed values^1^	Wheat	Soybean meal	Sorghum
Dry matter	87.5	90.1	87.2
AMEn, kcal/kg	3,067	2,285	3,208
Crude protein	10.0	46.9	9.21
Crude fat	1.59	1.28	3.10
Crude fiber	2.55	4.24	2.25
Digestible Arg	0.460	3.13	0.320
Digestible Lys	0.260	2.62	0.190
Digestible Met	0.140	0.600	0.140
Digestible Trp	0.130	0.620	0.090
Digestible Ile	0.290	2.01	0.300
Digestible Thr	0.260	1.69	0.250
Digestible Val	0.370	1.97	0.380
Total phosphorus	0.290	0.690	0.290
Available phosphorus	0.170	0.250	0.070
Ash	1.46	6.36	1.42

AMEn = apparent metabolisable energy, nitrogen-corrected.

1 Ingredients were analysed using near-infrared spectroscopy (NIR).

### Necrotic enteritis challenge

The NE challenge model was applied in the present study referencing the previous reports (Wu, et al., 2014; Rodgers, et al., 2015), where live sporulated oocysts of *Eimeria* strains were employed as a predisposing factor and *C. perfringens* as a primary causative agent to produce NE. In brief, on d 9, birds in the challenged groups were orally inoculated with 1 mL *Eimeria* spp. containing 5000 oocysts of both *E. acervulina* and *E. maxima*, and 2500 oocysts of *E. brunetti* (Eimeria Pty Ltd., Ringwood, VIC, Australia). On d 14 and 15, birds in the challenged groups were orally inoculated with 1 mL *C. perfringens* (EHE-NE18) consisting of approximately 10^8^ CFU/mL (CSIRO Livestock, Geelong, VIC, Australia). Concurrently, birds in the non-challenged group were orally administered with 1 mL phosphate-buffered saline (**PBS**) on d 9 and sterile thioglycolate broth on d 14 and 15.

### Performance measurement and sampling

Bird weight (**BW**) and feed intake were recorded per pen on d 0, 8, 19, 28 and 35 and average weight gain (**AWG**), average feed intake (**AFI**), and feed conversion ratio (**FCR**) in each feeding phase were subsequently calculated. Feed intake was calculated based on the dry matter (**DM**) content of the feed (feed in and feed out). The FCR was also calculated on a DM basis. Both the AFI and FCR were presented on an 88 % DM basis (Noblet, et al., 2015; Khairunnesa, et al., 2025). On d 35, individual BW of birds was taken to assess flock uniformity. The coefficient of variation (**CV**) of BW of birds in each treatment group was calculated to determine flock uniformity. Mortality and culled birds were recorded daily and the BW of dead and culled birds were recorded to adjust AFI and FCR accordingly. A necropsy was conducted to determine the cause of death. All the dead, culled, sampled and remaining birds on d 35 were examined to determine the sex by visual observation of testes.

At the end of 35-d experimental period, the production efficiency factor (**PEF**) was calculated using the previously described formula (Huff, et al., 2013):

$$\text{PEF}=\;\frac{\text{Body}\;\text{weight}\;\left(\text{kg}\right)\;\text{on}\;d\;35\;\times \;\text{Liveability}\;\text{on}\;d\;35\;\left(\%\right)}{\text{Age}\;\text{in}\;\text{days}\;\times \;\text{FCR}\;\left(d\;0-35\right)}\;\times \;100$$

On d 16, four randomly selected birds from each pen were weighed, electrically stunned by using an electric stunner (JF poultry equipment, Weltevreden Park, South Africa) and decapitated for blood collection. Birds were euthanised, blood and tissue samples were taken and stored at appropriate temperatures for further laboratory measurements.

### Intestinal lesion scoring

Four sampled birds per pen were scored for NE-caused intestinal lesions in the duodenum and jejunum by visual observation using a previously established lesion scoring system (Keyburn, et al., 2006; Shojadoost, et al., 2012). The 7-point scale lesion scoring system was used, where 0 indicated the absence of lesions and 6 denoted the most severe macroscopic lesions.

### Serum FITC-d measurement

On d 16, two birds from each pen were randomly selected, weighed, colour-marked and inoculated with 1 mL per os fluorescein isothiocyanate dextran (**FITC-d**; average molecular weight:4,000, FITC:Glucose = 1:250, Sigma-Aldrich Co., Missouri, USA) containing 4.17 mg/kg, approximately 2.5 h before euthanasia. These birds were returned to the pens where they were initially. After approximately 2.5 h, blood samples were collected for FITC-d determination in serum. Samples were kept at room temperature for approximately 3 h to allow clotting, centrifuged at 3,000 × *g* for 10 min to separate serum from whole blood, and immediately stored at −20°C until measurements were performed. Fluorescence concentrations of diluted serum (1:1 in PBS) were measured at an excitation wavelength of 485 nm and an emission wavelength of 528 nm using a multi-mode microplate reader, Synergy HT (SpectraMax M2e, Molecular Devices, San Jose, USA) and FITC-d concentration per mL of serum was calculated from a standard curve established with a known concentration of FITC-d and expressed as µg/mL.

### ELISA analyses

The total antibody titre concentrations of immunoglobulin A (**IgA**), M (**IgM**) and Y (**IgY**) in serum samples collected from one bird per pen on d 16 were measured using ELISA assays. Serum IgA, IgM and IgY concentrations were determined using chicken-specific ELISA reagents in accordance with the manufacturer's guidelines (Abnova chicken ELISA assay kits, Taipei City, 114 Taiwan). Antibody concentrations were determined using the standard curve constructed with a known concentration and expressed as mg/mL.

### Jejunal histomorphology

Proximal jejunal tissues collected from one bird per pen for intestinal histo-morphology were sectioned (4 μm) and processed using the standard Haematoxylin and Eosin assay as previously reported (Golder, et al., 2011). Villus height (**VH**) and crypt depth (**CD**) were measured with a minimum of 10 villi per pen and associated crypts randomly chosen for measurements. Histology slides were scanned (Hamamatsu Photonics K.K., Higashi-ku, Hamamatsu city, 431-3196, Japan) and parameters were measured using NDP.view 2.5 software (Hamamatsu Photonics K.K., Higashi-ku, Hamamatsu city, 431-3196, Japan).

### RNA extraction and cDNA synthesis

Proximal jejunal tissue samples were collected from one bird per pen on d 16 and total RNA was extracted from each tissue sample and purified using the RNeasy Mini Kit (Qiagen, Hilden, Germany) following the manufacturer’s instructions. The purity and quantity of total RNA samples were assessed using a NanoDrop ND-8000 spectrophotometer (Thermo Fisher Scientific, Waltham, USA). An RNA 6000 Nano kit was used to measure RNA integrity number (**RIN**) with the Agilent 2100 Bioanalyzer (Agilent Technologies, Inc., Waldbronn, Germany). Purified RNA samples were deemed to have high integrity if their RIN number exceeded 7.0. The purified RNA samples were reverse-transcribed using the QuantiTect Reverse Transcription Kit (Qiagen, Hilden, Germany) following the manufacturer’s guidelines. In summary, one µg of each total RNA sample was incubated at 42°C for 2 minutes in 2 µl of 7 × genomic DNA (**gDNA**) Wipeout Buffer to prevent gDNA contamination. Subsequently, the gDNA elimination reaction was incorporated into reverse-transcription reaction components, which comprised one µl of Quantiscript Reverse Transcriptase, 4 µl of 7 × Quantiscript RT Buffer, and one µl of RT Primer Mix and mixed properly. The Rotorgene Q real-time PCR machine (Rotor-Gene Q, QIAGEN GmbH, Hilden, Germany) was used to incubate the mixture at 42°C for 15 minutes followed by 95°C for 3 minutes to synthesise cDNA from the RNA. The cDNA samples were subsequently diluted tenfold with Nuclease-free water and stored at -20°C for further laboratory analysis.

### Real-time quantitative polymerase chain reaction (RT-qPCR)

Detection and amplification of genes were conducted in duplicates using a SYBR Green kit SensiFAST™ SYBR® No-ROX (Bioline, Sydney, Australia) with a Rotorgene Q real-time PCR machine (Rotor-Gene Q, QIAGEN GmbH, Hilden, Germany). The PCR reaction was performed in a volume of 10 µL comprising 2 µL of 10 × diluted cDNA template, 400 mM of each primer, and 5 µL of 2 × SensiFAST™ SYBR® No-ROX. A total of 8 house-keeping genes, namely, glyceraldehyde-3-Phosphate dehydrogenase (***GAPDH***), tyrosine 3- monooxygenase/tryptophan 5- monooxygenase activation protein zeta (***YWHAZ***), ribosomal protein L4 (***RPL4***), beta actin (***ACTB***), hydroxymethylbilane synthase (***HMBS***), Hypoxanthine phosphoribosyltransferase 1 (***HPRT1***), succinate dehydrogenase subunit A (***SDHA***), and TATA-Box Binding Protein (***TBP***) were selected for the optimisation of reference genes applying the gene expression stability measure (geNorm M) module in qbase+ software version 3.0 (Biogazelle, Zwijnbeke, Belgium). The two most stable housekeeping genes with the lowest M-value (< 0.5), *HMBS* and *RPL4* were selected as optimised reference genes to normalise the expression of the target genes. The amplification cycle (**Cq**) values for candidate target genes were gathered and imported into qBase+ version 3.0 software (Biogazelle, Zwijnbeke, Belgium) for analysis against the reference genes. The qbase+ used the arithmetic mean approach to convert logarithmic Cq values to linear relative quantities by applying the exponential function for relative quantification of genes (Vandesompele, et al., 2002; Hellemans, et al., 2007) and the resulting data were exported for the statistical analysis. The normalised relative quantities (**NRQ**) values were computed and assessed across all samples corresponding to each target gene. The primers used in this study were either obtained from previously published studies in chickens or designed using the NCBI Primer-BLAST tool (https://www.ncbi.nlm.nih.gov/tools/primer-blast/) as shown in Table 4. Notably, this work corrects a typographical error in the reverse primer sequence for *CASP8* that was reported in our previous study (Gharib-Naseri et al., 2021). Agilent 2100 Bioanalyzer (Agilent Technologies, Inc., Waldron, Germany) was employed to test the specificity of each pair of primers before qPCR analysis using Agilent DNA 1000 Kit (Agilent Technologies, Inc., Waldron, Germany), and only primers that specifically amplified target fragments were used in the qPCR assay.

**Table 4:** Sequences of primers used for quantitative real-time PCR.

Item	Sequence	Size (pb)	Annealing T°	Reference
*TJP1*	F-GGATGTTTATTTGGGCGGC R-GTCACCGTGTGTTGTTCCCAT	187	60	Gharib-Naseri, et al. (2021)
*OCLN*	F- ACGGCAGCACCTACCTCAA R- GGGCGAAGAAGCAGATGAG	123	60	Du, et al. (2016)
*JAM2*	F-AGACAGGAACAGGCAGTGCTAG R-ATCCAATCCCATTTGAGGCTAC	135	60	Kumar, et al. (2021)
*CASP3*	F-TGGTGGAGGTGGAGGAGC R- GTTTCTCTGTATCTTGAAGCACCA	110	62	Gharib-Naseri, et al. (2021)
*CASP8*	F- GGAGCTGCTCTATCGGATCAAT R- AGCAGATACCTGAACGGAGACAC	126	60	Gharib-Naseri, et al. (2021)
**Reference genes**				
*HMBS*	F: GGCTGGGAGAATCGCATAGG R: TCCTGCAGGGCAGATACCAT	66	61	Yin, et al. (2011)
*RPL4*	F: TTATGCCATCTGTTCTGCC R: GCGATTCCTCATCTTACCCT	235	60	Yang, et al. (2013)

### Litter moisture, litter, footpad dermatitis and hock burn scores

On d 35, litter structure (quality) per pen was evaluated through a visual examination using a scoring system ranging from 0 to 3 according to Kheravii, et al. (2017). The 4-point scales were established as follows: 0 = dry litter; 1 = slightly caked /moist litter; 2 = more caked/ moist litter; 3 = wet litter. Visual examination was performed at four different locations within each pen and the average was calculated to determine the litter scores for each pen. On the same day, approximately one Kg of pooled litter content was collected into plastic bags from six different points in each pen, including around the feeder, drinkers, and the beginning of the pen. The fresh weight of litter content and its dry weight obtained after drying litter in a forced air oven at 105°C for 24 hours were recorded. The litter moisture content was measured and calculated using the method described previously Barker, et al. (2013).

On d 35, all individual birds in each pen were visually examined and scored for footpad dermatitis (**FPD**) and hock burn (**HB**) following the scoring method outlined in the Welfare Quality® Assessment protocol for poultry (Quality, 2009). A 5-point scoring method was used to examine the severity and appearance of lesions in the footpad and hock, where 0 indicated no lesions and 4 represented the most severe macroscopic lesions. The visual scoring was conducted by two experienced researchers blinded to the experimental design and pen arrangements.

### Data analysis

The data generated in this study were checked for normal distribution before statistical analysis. The performance data were analysed as a completely randomised design using JMP® 18.0 (SAS Institute, Cary, NC, USA), where the pen served as an experimental unit (*n* = 32). The significant differences between means were separated by Tukey’s test. Performance data were analysed for the treatment effects, with the female percentage included as a covariate when significant. The intestinal lesion, footpad dermatitis and hock burn scores were analysed using the nonparametric Kruskal-Wallis test as the data were not normally distributed. The means were considered significantly different when *P*-value was < 0.05, and declared a tendency to be different with 0.05 < *P* < 0.10. Additionally, outcomes were described as ‘shifting’ when they exhibited a transitional or intermediate response between treatment groups, demonstrating no significant difference from both control groups (which were significantly different) but indicating an effect of the additive treatment compared to the negative control, where the additive was supplemented.

## Results

### Performance, production efficiency factor and flock uniformity

**Fig. 1:**
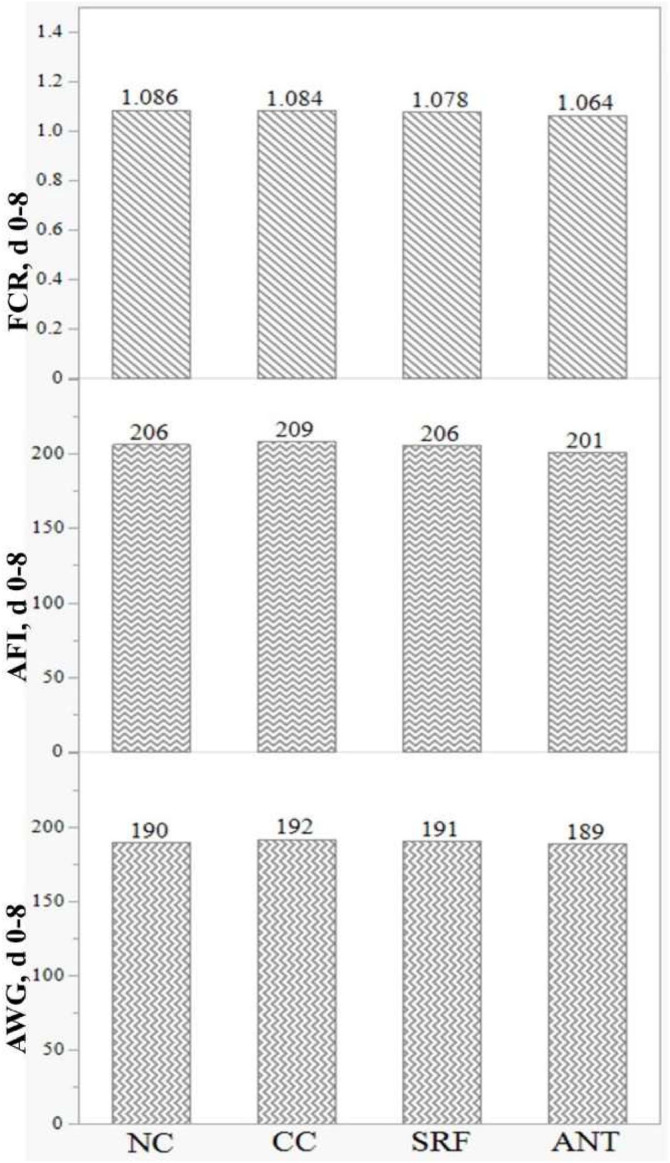
Effects of SRF on performance of broilers before the challenge (d 0-8). AWG = average weight gain (*P* = 0.736); AFI = average feed intake (*P* = 0.339); FCR = feed conversion ratio (*P* = 0.277). The FI and FCR values were standardised for 88 % dry matter. NC, Non-challenged control; CC, Challenged control; SRF, Surfactin; ANT, Antibiotics (Zn bacitracin and Salinomycin).

In the starter phase (d 0-8), i.e., before the NE challenge, AWG, AFI and FCR were not different among the treatments (*P* > 0.05) as shown in Fig. 1.

The effects of SRF and NE challenge on the performance of broilers at different phases are shown in Table 5. Overall, one-way ANOVA analysis revealed significant differences in the following measurements: AWG from d 8-19 (*P* < 0.001), d 19-28 (*P* = 0.006) and d 0-35 (*P* < 0.001), AFI from d 8-19 (*P* < 0.001) and d 19-28 (*P* = 0.031), FCR from d 8-19 (*P* < 0.001), d 19-28 (*P* = 0.031), d 28-35 (*P* = 0.008), d 19-35 (*P* = 0.006) and d 0-35 (*P* < 0.001), and liveability from d 19-28 (*P* = 0.015).

**Table 5:** Effects of SRF and NE challenge on the performance in broilers at different phases.

Treatment^1^	NC	NE challenged^2^			SEM^3^	*P*-value
		CC	SRF	ANT		
**d 8-19**						
AWG, g	718^b^	648^c^	647^c^	771^a^	11	<0.001
AFI, g	937^b^	911^b^	920^b^	1014^a^	12	<0.001
FCR	1.304^b^	1.407^a^	1.422^a^	1.315^b^	0.010	<0.001
Liveability %	99.2	96.8	98.4	98.4	1.0	0.457
**d 19-28**						
AWG, g	930^ab^	894^b^	886^b^	940^a^	12	0.006
AFI, g	1385^ab^	1351^b^	1355^b^	1402^a^	12	0.031
FCR	1.490^b^	1.512^ab^	1.531^a^	1.491^b^	0.009	0.031
Liveability %	96.7^b^	100^a^	100^a^	100^a^	0.8	0.015
**d 28-35**						
AWG, g	783	741	784	746	16	0.104
AFI, g	1333	1358	1347	1291	22	0.284
FCR	1.705^b^	1.833^a^	1.719^b^	1.734^ab^	0.024	0.008
Liveability %	99.0	96.7	96.8	95.8	1.9	0.672
**d 19-35**						
AWG, g	1713	1632	1673	1685	22	0.102
AFI, g	2715	2696	2700	2687	27	0.931
FCR	1.585^b^	1.652^a^	1.615^ab^	1.595^b^	0.011	0.006
Liveability %	95.6	96.7	96.8	95.8	2.0	0.966
**d 0-35**						
AWG, g	2620^ab^	2475^c^	2513^bc^	2644^a^	30	<0.001
AFI, g	3791	3763	3782	3840	36	0.604
FCR	1.448^b^	1.521^a^	1.505^a^	1.453^b^	0.008	<0.001
Liveability %	95.3	94.5	96.1	95.3	1.9	0.949

AWG = average weight gain; AFI = average feed intake; FCR = feed conversion ratio; NE = necrotic enteritis; FI and FCR values were standardised for 88 % dry matter.

1 NC, Non-challenged control; CC, Challenged control; SRF, Surfactin; ANT, Antibiotics (Zn bacitracin and Salinomycin).

2 Challenged birds were orally gavaged with *Eimeria* spp. on d 9 and *Clostridium perfringens* on d 14 and 15.

3 SEM: standard error of means.

a – c Values in a row with no common superscripts differ significantly (*P* < 0.05).

In the grower phase (d 8-19), the challenge significantly decreased AWG and increased FCR in the CC group compared to the NC group (*P* < 0.05). Birds fed ANT had a higher AWG and AFI, and lower FCR compared to the CC (*P* < 0.05). The inclusion of SRF did not affect AWG, FI and FCR compared to the CC group (*P* > 0.05).

During d 19-28, birds fed ANT had an improved (*P* < 0.05) AWG and FCR compared to the CC group, whereas the NC group was in between them (*P* > 0.05). Supplementation of SRF did not affect performance parameters compared to the CC group (*P* > 0.05). Liveability was lower in the NC group compared to other treatment groups (*P* < 0.05). During d 28-35, the effects of NE challenge on AWG and AFI were not observed (*P* > 0.05). Birds fed SRF had a lower FCR compared to the CC group (*P* < 0.05). Throughout the finisher phase (d 19-35), the CC group had a higher FCR compared to the NC group (*P* < 0.05), while supplementation of SRF shifted the FCR from the CC group towards the NC and ANT groups (*P* > 0.05).

**Fig. 2:**
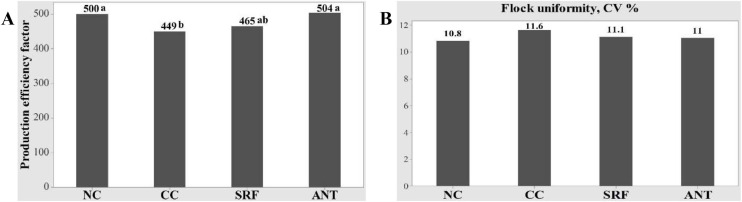
Effects of SRF and NE challenge on production efficiency and flock uniformity of broilers. A) PEF = production efficiency factor (*P* = 0.003); B) Flock uniformity based on CV%; NE = necrotic enteritis. NC, Non-challenged control; CC, Challenged control; SRF, Surfactin; ANT, Antibiotics (Zn bacitracin and Salinomycin). ^a,b^Values within a column with different letters differ significantly (*P* < 0.05).

During the overall period of study (d 0-35), the NE challenge significantly decreased AWG and increased FCR in the CC group compared to the NC group (*P* < 0.05). Birds fed ANT had a higher AWG and lower FCR compared to the CC group (*P* < 0.05). Birds fed SRF had a statistically similar AWG compared to the CC and NC groups, indicating a shift of AWG from CC towards NC group due to the addition of SRF (*P* > 0.05). Liveability and AFI were not different among the treatment groups (*P* > 0.05). The CC group had a lower PEF compared to the NC group (*P* < 0.05), while supplementation of SRF shifted the PEF from the CC group towards the NC and ANT groups (*P* > 0.05; Fig. 2**A**).

The effects of NE challenge and dietary inclusion of SRF on flock uniformity on d 35 are shown in Fig. 2**(B)**. The CV (%) of BW followed the following order across the treatment groups: NC <ANT < SRF < CC. The NC group had the lowest CV, while the CC group had the highest CV, and ANT and SRF groups were intermediate.

### Intestinal lesions

**Fig. 3:**
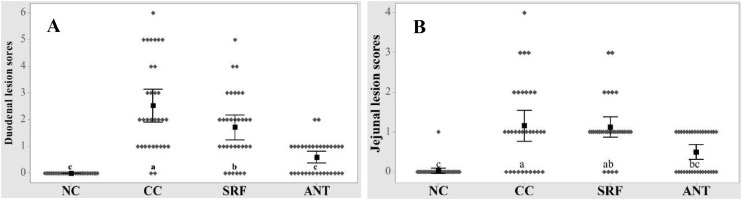
Effects of SRF and NE challenge on intestinal lesions of broilers on d 16. A) Duodenal lesion scores, *P* < 0.001; B) Jejunal lesion scores, *P* < 0.001; NE = necrotic enteritis. NC, Non-challenged control; CC, Challenged control; SRF, Surfactin; ANT, Antibiotics (Zn bacitracin and Salinomycin). ^a-c^Values within a column with different letters differ significantly (*P* < 0.05).

The effects of NE challenge and SRF on duodenal and jejunal lesion scores on d 16 are presented in Fig. 3**A and B**, respectively. The non-parametric Kruskal-Wallis test indicated that the intestinal lesion scores in the duodenum (*P* < 0.001) and jejunum (*P* < 0.001) were significantly different. The NE challenge significantly increased duodenal and jejunal lesion scores in the CC group compared to the NC group (*P* < 0.05). Birds fed ANT had lower duodenal and jejunal lesion scores compared to the CC group (*P* < 0.05). Birds fed SRF had a significantly lower lesion score in the duodenum only, compared to the CC group (*P* < 0.05). Birds fed SRF had no differences in jejunal lesion scores compared to the CC and ANT groups, showing a shift from the CC group to the ANT group (*P* > 0.05).

### Serum FITC-d concentration and immunoglobulins

The effects of SRF and NE challenge on serum FITC-d concentration and immunoglobulins of broilers on d 16 are shown in Table 6. One-way ANOVA analysis showed significant differences in serum FITC-d (*P* < 0.001), IgA (*P* = 0.031), IgM (*P* = 0.001), and IgY (*P* = 0.036).

**Table 6:** Effects of SRF and NE challenge on the FITC-d concentrations and immunoglobulins in broilers on d 16.

Treatment^1^	NC	NE challenged^2^			SEM^3^	*P*-value
		CC	SRF	ANT		
**Serum FITC-d** **(µg/mL)**	0.036^c^	0.181^a^	0.139^a^^b^	0.058^b^^c^	0.026	<0.001
**Serum immunoglobulins (mg/mL)**						
IgA	0.208^a^	0.128^ab^	0.190^ab^	0.105^b^	0.027	0.031
IgM	0.126^a^	0.059^b^	0.070^b^	0.055^b^	0.013	0.001
IgY	1.61^a^	0.847^ab^	0.706^b^	0.829^ab^	0.227	0.036

FITC-*d* = fluroscent isothiocynate dextran; NE = necrotic enteritis;.

1 NC, Non-challenged control; CC, Challenged control; SRF, Surfactin; ANT, Antibiotics (Zn bacitracin and Salinomycin).

2 Challenged birds were orally gavaged with *Eimeria* spp. on d 9 and *Clostridium perfringens* on d 14 and 15.

3 SEM: standard error of means.

a – c Values in a row with no common superscripts differ significantly (*P* < 0.05).

The NE challenge significantly increased serum FITC-d concentration in the CC group compared to the NC group (*P* < 0.05). Birds fed ANT had lower FITC-d concentrations compared to the CC group (*P* < 0.05). Birds fed SRF had intermediate FITC-d concentration, namely statistically no differences from the CC and ANT groups (*P* > 0.05), indicating a shift of the value towards the ANT group. The NE challenge significantly reduced serum IgM level in the CC group compared to the NC group (*P* < 0.05). Birds fed ANT had lower IgA and IgM levels than the NC group (*P* < 0.05), whereas IgA and IgM levels did not differ between CC, SRF and ANT groups (*P* > 0.05). Birds fed SRF had a lower IgY compared to the NC group (*P* < 0.05) but were not different from the CC and ANT groups (*P* > 0.05).

### Jejunal gene expressions and histomorphology

The effects of SRF and NE challenge on jejunal gene expressions and histomorphology of broilers on d 16 are shown in Table 7. One-way ANOVA analysis showed that the expression of *OCLN* (*P* < 0.001) and *TJP1* (*P* = 0.001) genes, CD (*P* < 0.001) and VH:CD (*P* < 0.001) in the jejunum were significantly different, whereas VH (*P* = 0.062) showed a tendency toward significance.

**Table 7:** Effects of SRF and NE challenge on jejunal gene expressions and histomorphology in broilers on d 16.

Treatment^1^	NC		NE challenge^2^			SEM^3^	*P*-value
			CC	SRF	ANT		
**Gene expressions**							
*OCLN*	2.11^a^		0.77^b^	0.85^b^	0.91^b^	0.14	<0.001
*TJP1*		1.36^a^	0.85^b^	0.99^b^	0.95^b^	0.08	0.001
*JAM2*	1.29		1.11	1.22	0.90	0.19	0.518
*CASP3*	1.20		1.25	1.28	0.74	0.17	0.096
*CASP8*	1.02		1.14	1.10	0.88	0.09	0.230
**Histomorphology**							
VH, µm	1698		1517	1733	1754	65	0.062
CD, µm	157^c^		305^a^	230^b^	199^bc^	15	<0.001
VH:CD	11.0^a^		5.00^c^	7.90^b^	9.28^ab^	0.65	<0.001

VH = villus height; CD = crypt depth; VH:CD = villus height and crypt depth ratio; NE = necrotic enteritis.

1 NC, Non-challenged control; CC, Challenged control; SRF, Surfactin; ANT, Antibiotics (Zn bacitracin and Salinomycin).

2 Challenged birds were orally gavaged with *Eimeria* spp. on d 9 and *Clostridium perfringens* on d 14 and 15.

3 SEM: standard error of means.

a,b Values in a row with no common superscripts differ significantly (*P* < 0.05).

The NE challenge downregulated the expression of *OCLN* and *TJP1* in the CC group compared to the NC group (*P* < 0.05). The expressions of these genes were not different between CC, SRF and ANT groups (*P* > 0.05). The expressions of *JAM2, CASP3* and *CASP8* were not different among the treatment groups (*P* > 0.05). The NE challenge significantly increased CD and reduced VH:CD in the CC group compared to the NC group (*P* < 0.05). Birds fed SRF had significantly lower CD and higher VH:CD compared to the CC group (*P* < 0.05). The VH was not significantly different among the treatment groups, but there was a tendency (0.05 < *P* < 0.10). The ANT group had the highest VH (1754 µm), and the CC group had the lowest VH (1517 µm), while NC and SRF groups were intermediate (1698 µm and 1733 µm, respectively).

### Litter quality and footpad health

The effects of SRF and NE challenge on litter quality and footpad health of broilers on d 16 are shown in Table 8. One-way ANOVA analysis showed significant differences in litter moisture (*P* = 0.030), litter score (*P* < 0.001), HB score (*P* < 0.001), and FPD score (*P* = 0.003). The NE challenge significantly increased litter moisture, litter scores, HB and FPD scores in the CC group compared to the NC group (*P* < 0.05). However, dietary supplementation of SRF shifted the litter moisture content from the CC group towards the NC and ANT groups, and litter and HB scores towards those of the ANT group (*P* > 0.05). Furthermore, the inclusion of SRF significantly reduced FPD scores compared to the CC group (*P* < 0.05).

**Table 8:** Effects of SRF and NE challenge on litter quality and footpad health in broilers on d 35.

Treatment^1^	NC		NE challenge			SEM^2^	*P*-value
			CC	SRF	ANT		
Litter moisture	27.4^b^		31.2^a^	28.5^ab^	28.5^a^^b^	0.9	0.030
Litter score		1.25^c^	1.88^a^	1.59^ab^	1.41^bc^	0.12	<0.001
HB score	0.500^c^		1.80^a^	1.37^ab^	1.09^b^	0.12	<0.001
FPD score	0.043^b^		0.526^a^	0.142^b^	0.239^ab^	0.086	0.003

FPD = Footpad dermatitis; HB = Hock burn; NE = necrotic enteritis.

1 NC, Non-challenged control; CC, Challenged control; SRF, Surfactin; ANT, Antibiotics (Zn bacitracin and Salinomycin).

2 Challenged birds were orally gavaged with *Eimeria* spp. on d 9 and *Clostridium perfringens* on d 14 and 15. ^3^SEM: standard error of means.

a – c Values in a row with no common superscripts differ significantly (*P* < 0.05).

## Discussion

With the removal of in-feed antibiotics in poultry, alternatives to antibiotics have been sought to prevent and control NE in broilers. The current study examined the potential of SRF to alleviate the adverse impacts of NE on performance, intestinal health and bird welfare parameters in broilers. The reduced AWG, increased FCR, more severe intestinal lesions, higher serum FTIC-d concentrations, enhanced CD and reduced VH:CD in the jejunum observed in NE challenged control broilers indicate the successful induction of the challenge. Furthermore, ANT-treated birds demonstrated the protective effects against NE as demonstrated by improved AWG and FCR, reduced serum FTIC-d concentrations and enhanced jejunal VH:CD. The findings from the present study showed that SRF significantly reduced FCR in the post-challenge recovery phase, reduced duodenal lesion scores and jejunal CD, increased jejunal VH:CD, and decreased FPD scores compared to the birds in the CC group. SRF also shifted the FCR in the finisher phase, AWG and PEF in an overall period, serum FTIC-d concentrations, jejunal lesion scores, flock uniformity, HB and litter scores, and litter moisture from the CC group towards the levels closer to the NC and ANT groups. The results of this study support the hypothesis that dietary inclusion of SRF helps to reduce the negative impacts of NE in intestinal health through different mechanisms and is able to restore the performance and enhance bird welfare parameters.

Antibacterial peptides produced by *B. subtilis* demonstrate broad-spectrum efficacy against pathogenic microorganisms. Among these, SRF, a cyclic lipopeptide produced by *Bacillus*, is a notable biosurfactant with diverse antibacterial properties (Chen, et al., 2008; Sumi, et al., 2015). The mechanism by which antimicrobial peptides inhibit microbial growth is not fully understood. However, a recent review (Chen, et al., 2022) highlighted that, the antibacterial effects of SRF are attributed to the following mechanisms: 1) disrupting the cell membrane of pathogenic bacteria, leading to osmotic pressure imbalance or membrane disintegration; 2) inhibiting protein synthesis in pathogenic bacteria, hence preventing cellular reproduction; 3) inhibiting enzyme activity in pathogenic bacteria, which disrupts normal cellular metabolism. It is also apparent that surfactin exhibits several properties commonly associated with probiotics. This can be attributed to its ability to regulate intestinal flora through antibacterial properties, which enhance digestive enzyme activity and promote intestinal homeostasis and overall health. A previous study showed the beneficial effects of SRF under a cyclophosphamide-induced gut dysbiosis model in mice, where SRF acts as an immunomodulator by enhancing immune cell efficacy, modifying gut microbiota composition and mitigating weight loss and intestinal inflammation (Jia, et al., 2024). The results observed in the current study indicated that dietary inclusion of SRF shifted the FCR in the finisher phase, AWG and PEF in an overall period from the CC group towards the levels closer to NC and ANT groups. Birds fed SRF had higher BW uniformity on day 35, indicating better flock uniformity. In addition, birds fed SRF significantly improved the FCR during the finisher phase (d 28-35), which shows the beneficial effects of SRF supplementation in diets in the post-challenge recovery period. Similar to the findings of this study, dietary supplemented fermented product of *B. subtilis* enriched with SRF at different doses improved AWG, AFI and PEF over the entire study period under normal feeding conditions (Lee, et al., 2023, 2024) or upon NE challenge (Cheng, et al., 2018). The enhanced feed efficiency, AWG and PEF in the SRF supplemented group are likely attributable to improved intestinal health as evidenced by reduced intestinal lesions and jejunal CD, increased VH:CD and also a trend to reduce serum FITC-d concentrations as an indicative intestinal integrity in this study.

A healthy intestinal mucosa is crucial for efficient nutrient digestion and absorption and serves as a protective barrier against pathogenic bacterial infections (Balda and Matter, 2008; Sánchez de Medina, et al., 2014). Studies have shown that enteric inflammation compromises the intestinal mucosa and tight junctions, leading to increased intestinal permeability (Vicuña, et al., 2015; Barekatain, et al., 2019). In this study, birds challenged with NE displayed higher serum FITC-d concentrations compared to the NC group on d 16, indicating compromised gut integrity caused by NE. Thus, the trend to reduce serum FITC-d concentrations in the SRF-supplemented group demonstrated the beneficial effects of its dietary inclusion on intestinal integrity in birds exposed to *Eimeria* spp. and *C. perfringens*. Additionally, birds fed SRF showed higher, although numerically, serum IgA and IgM on d 16, during the onset of NE challenge, supporting the immunomodulatory effects of SRF supplementation in diets, which may have contributed to the faster recovery of birds from the challenge, ultimately improving the FCR in later phases. It is well-established that wet or poor-quality litter is highly correlated with NE (Williams, 2005; Sharma, et al., 2018), and wet litter significantly increases the risk of FPD (Cengiz, et al., 2011; Kheravii, et al., 2017). Therefore, it can be speculated that improved feed efficiency in the finisher phase and reduced NE effects on intestinal health might positively influenced litter quality and footpad health observed in this study. Moreover, higher VH and VH:CD are good indicators of a healthy intestine, supporting better digestion and nutrient absorption, which in turn enhances feed efficiency and growth ultimately contributing to improved litter quality and better footpad health. Collectively, these findings suggested that supplementation of SRF supported the performance and reduced the negative effects of NE on intestinal health. However, although SRF supplementation in diets at 0.01 % improved the intestinal health during the challenge period and performance in the later finisher phase which may have contributed to the overall performance improvement, SRF had no effects on performance in the starter and grower phases, particularly during the onset of NE. Further studies using different doses are needed to clarify this observation and to investigate the mechanisms by which SRF improves the performance and intestinal health.

The current findings suggest that the supplementation of SRF to mitigate the adverse effects of NE challenge on intestinal health of broilers is even more obvious as shown by the reduced CD and increased VH:CD in the jejunum. In addition, the inclusion of SRF in broiler diets led to a tendency to increase VH. Greater CD correlates with an elevated cell turnover rate, resulting in increased energy consumption waste. Conversely, a reduced villus height correlates with a diminished surface area of the intestinal wall and fewer mature epithelial cells, hence impairing digestion and absorption. Thus, from a functional perspective, higher VH, lower CD and enhanced VH:CD are considered optimal, as they enhance nutrient absorption and maintain intestinal integrity (Montagne, et al., 2003), thereby improving growth performance. Similar findings were reported in a previous study under a cyclophosphamide immunosuppression model in mice (Jia, et al., 2024), where inclusion of SRF significantly improved VH and VH:CD, and decreased CD. This is further supported by another study showing that a diet containing a fermented product of *B. subtilis* enriched with SRF has the potential to improve growth performance and intestinal morphology under NE challenge conditions (Cheng, et al., 2018), which confirms the beneficial effects of SRF supplementation on intestinal health in broilers.

Litter quality and footpad health serve as important markers of bird management and health. These parameters also play a vital role in assessing the welfare status of birds, particularly when dietary additives are employed to alleviate the negative effects associated with NE. Previous studies have shown that various factors could significantly increase the litter moisture and simultaneously the prevalence of FPD in broiler flocks, including diets and ingredients (Youssef, et al., 2012; Cengiz, et al., 2013), litter materials and quality (Bilgili, et al., 2009; Kheravii, et al., 2017), enteric diseases (Kaldhusdal and Hofshagen, 1992; Sharma, et al., 2018), high stocking density and various management practices such as ventilation (Thaxton, et al., 2006). It has been shown that enteric diseases such as coccidiosis and NE cause intestinal damage and diarrhoea which contributes to increased litter moisture (Dunlop, et al., 2016; Daneshmand, et al., 2023). The main consequence of wet litter in poultry flocks is FPD, which is characterised by lesions on the plantar surface of the feet (Greene, et al., 1985). Thus, this disease is associated with the economic profits of the poultry flocks as it can reduce the bird movement, decline bird welfare and ultimately affect the performance. Furthermore, in many countries where chicken paws are highly sought-after edible parts, feet exhibiting severe dermatitis are deemed unsuitable for human consumption, leading to significant economic losses (Shepherd and Fairchild, 2010). In the current study, it is speculated that the reduced NE effects on intestinal health as indicated by improved intestinal integrity, immunity and histomorphology played a crucial role in lowering litter moisture and litter scores, which in turn, reduced FPD and HB scores observed in this study. The high litter quality, low FPD and HB scores further suggest that the dietary supplementation of SRF mitigated the occurrence of NE and enhanced bird health and welfare status.

In summary, the findings of this study suggest that dietary supplementation of SRF at 0.01 % has the potential to reduce the incidence of subclinical NE in broilers by improving intestinal integrity, villus structure and immunity, while reducing intestinal lesions. The results also suggest that SRF supplementation can maintain similar AWG compared to the NC group, PEF in the overall study period comparable to that of the NC and ANT fed birds and improve bird welfare. Evidently, SRF inclusion in the diet significantly reduced FCR in the post-challenge recovery phase, duodenal lesion scores and jejunal CD, enhanced jejunal VH:CD, and decreased FPD scores. Further, SRF supplementation positively shifted other critical parameters such as FCR in the finisher phase, AWG and PEF over the entire period, serum FTIC-d concentrations, jejunal lesion scores, flock uniformity, HB and litter scores and moisture from the CC group toward values observed in the NC and ANT groups. However, further research on SRF supplementation at different dosages is needed to better understand the mechanisms of action in enhancing intestinal health and subsequent performance, particularly during the onset of NE, which will provide valuable insights for the poultry industry in mitigating the challenges of antibiotic-free production.

## CRediT authorship contribution statement

**Alip Kumar:** Writing – original draft, Visualization, Project administration, Methodology, Formal analysis, Data curation, Conceptualization. **Kosar Gharib-Naseri:** Writing – review & editing, Validation, Investigation, Funding acquisition, Data curation, Conceptualization. **Most Khairunnesa:** Writing – review & editing, Methodology, Investigation, Data curation. **Sosthene Musigwa:** Writing – review & editing, Methodology, Data curation. **Reza Barekatain:** Writing – review & editing, Methodology, Investigation. **Li Li:** Writing – review & editing, Validation, Methodology. **Peng Chen:** Writing – review & editing, Validation, Methodology. **Shu-Biao Wu:** Writing – review & editing, Validation, Supervision, Methodology, Investigation, Funding acquisition, Conceptualization.

## Disclosures

We declare that we have no financial and personal relationships with other people or organisations that can inappropriately influence our work, there is no professional or other personal interest of any nature or kind in any product, service and/or company that could be construed as influencing the content of this paper. All activities involving animal experimentation in the present research have been conducted according to the approval of the Animal Ethics Committee of the University of New England. All the listed authors satisfy the authorship criteria and have read and approved this manuscript.
